# The Practitioners' Bookshelf

**Published:** 1893-03-11

**Authors:** 


### THE PRACTITIONERS' BOOKSHELF.

### "QUAIN'S ELEMENTS OF ANATOMY."*

When a text-book has reached its tenth edition, a reviewer's task is a very light one: the fact that another edition has been called for is the best possible sign that the work is highly valued. The volume before us consists of 200 pages, and has been prepared entirely by Professor Schäger, and it is everywhere marked by that care in preparation and clearness of expression that are so characteristic of Mr. Schäger's work. In no other branch of the subject has our knowledge of anatomy been added to so much in recent years as in neurology, and everywhere this volume shows evidence of careful editing to bring it well up to date. There are 139 illustrations, many of them are new and all are admirable. The volume is worthy in every respect of the high position "Quain's Anatomy" has held for many years past.

*"Quain's Elements of Anatomy." Edited by Edward Albert Schäger, F.R.S., Professor of Physiology and Histology in University College, London, and George Dance Thane, Professor of Anatomy in University College, London. In three volumes. Vol. III. Part 1—The Spinal Cord and Brain. By Professor Schäger. (London: Longmans, Green, and Co., 1893.)

